# Effect of pH of H_2_O_2_ solutions on the morphology and wear resistance of human dental enamel: an AFM study

**DOI:** 10.1080/07853890.2021.1897311

**Published:** 2021-09-28

**Authors:** A. C. Branco, M. Polido, R. Colaço, C. G. Figueiredo-Pina, A. P. Serro

**Affiliations:** a Centro de Química Estrutural, Instituto Superior Técnico, Universidade de Lisboa, Lisboa, Portugal; b Centro de Investigação Interdisciplinar Egas Moniz (CiiEM), Egas Moniz Cooperativa de Ensino Superior, Caparica, Portugal; c IDMEC e Departamento de Engenharia Mecânica, Instituto Superior Técnico, Universidade de Lisboa, Lisboa, Portugal; d Centro de Física e Engenharia de Materiais Avançados, Instituto Superior Técnico, Universidade de Lisboa, Lisboa, Portugal; e CDP2T and Department of Mechanical Engineering, School of Technology of Setúbal, Instituto Politécnico de Setúbal, Setúbal, Portugal

## Abstract

**Introduction:**

Teeth whitening is a highly demanded aesthetic dental procedure to remove teeth stains and get a whiter and perfect smile. This type of treatment is usually performed by the application on the teeth surface of hydrogen or carbamide peroxides which diffuse through the external layer of the teeth, enamel [1]. Although whitening treatments improve teeth appearance, they can impair teeth health, due to the eventual degradation of the teeth tissues. This may lead to abnormal wear and weaken the tooth structure. The aim of this work is to study the effect of the pH of 30% H_2_O_2_ solutions on the surface of human enamel.

**Materials and methods:**

Human molars were collected for the study with informed consents of the patients. Teeth were bleached in 30% H_2_O_2_ solution with different pH values (2, 4 and 6). All samples were submitted to 11 bleaching sessions of 10 min each with alternate periods (2 min) of exposition to blue light. Microhardness, surface roughness and topography/morphology were characterised by microindentation tests, atomic force microscopy (AFM) and scanning electron microscopy (SEM), respectively, before and after the whitening treatments. AFM scratching tests (10 × 10 µm^2^) were performed, using a diamond probe. After the nanowear tests, topographical images of larger areas (20 × 20 µm^2^) which include the scratched squares were obtained with a silicon probe. The wear mechanisms were evaluated by SEM. Acceptance of the study protocol was obtained from a local ethics committee.

**Results:**

The most significant changes on enamel surface were obtained for pH = 2: the microhardness decreases, and the surface roughness significantly increases as confirmed by the SEM image analysis. A higher wear rate was observed for pH = 2 in the AFM scratching tests. SEM observation of the wear zone showed differences in the wear mechanisms, which depend on the pH.

**Discussion and Conclusions:**

Enamel microhardness, surface roughness and wear resistance are affected by the pH of the bleaching solution: pH = 2 led to the most adverse effects, which was attributed to hydroxyapatite demineralisation. The study of the effect of pH in the bleaching treatments is crucial to optimise this type of procedures and avoids enamel damage.
